# Allelic characterization and protein structure analysis reveals the involvement of splice site mutation for growth habit differences in *Lablab purpureus* (L.) Sweet

**DOI:** 10.1186/s43141-021-00136-z

**Published:** 2021-02-22

**Authors:** Supriya Kaldate, Apexa Patel, Kaushal Modha, Vipulkumar Parekh, Bhushan Kale, Gopal Vadodariya, Ritesh Patel

**Affiliations:** 1 Department of Genetics and Plant Breeding, N. M. College of Agriculture, Navsari Agricultural University, Navsari, Gujarat 396 450 India; 2 Department of Basic Science and Humanities, ASPEE College of Horticulture and Forestry, NAU, Navsari, Gujarat 396 450 India

**Keywords:** Terminal flowering locus (*TFL*), Transition, Single-nucleotide polymorphism, Candidate gene approach

## Abstract

**Background:**

Interrelationship between growth habit and flowering played a key role in the domestication history of pulses; however, the actual genes responsible for these traits have not been identified in Indian bean. Determinate growth habit is desirable due to its early flowering, photo-insensitivity, synchronous pod maturity, ease in manual harvesting and short crop duration. The present study aimed to identify, characterize and validate the gene responsible for growth habit by using a candidate gene approach coupled with sequencing, multiple sequence alignment, protein structure prediction and binding pocket analysis.

**Results:**

Terminal flowering locus was amplified from GPKH 120 (indeterminate) and GNIB-21 (determinate) using the primers designed from *PvTFL1y* locus of common bean. Gene prediction revealed that the length of the third and fourth exons differed between the two alleles. Allelic sequence comparison indicated a transition from guanine to adenine at the end of the third exon in GNIB 21. This splice site single-nucleotide polymorphism (SNP) was validated in germplasm lines by sequencing. Protein structure analysis indicated involvement of two binding pockets for interaction of terminal flowering locus (*TFL*) protein with other proteins.

**Conclusion:**

The splice site SNP present at the end of the third exon of *TFL* locus is responsible for the transformation of shoot apical meristem into a reproductive fate in the determinate genotype GNIB 21. The splice site SNP leads to absence of 14 amino acids in mutant *TFL* protein of GNIB 21, rendering the protein non-functional. This deletion disturbed previously reported anion-binding pocket and secondary binding pocket due to displacement of small β-sheet away from an external loop. This finding may enable the modulation of growth habit in Indian bean and other pulse crops through genome editing.

## Background

Indian bean (*Lablab purpureus* (L.) Sweet) is a short-day vegetable as well as a split pulse crop of tropical countries, viz., India and Africa. It is also utilized as cover and forage crops especially in drought-prone areas due to its inherent drought tolerance. It also fixes atmospheric nitrogen, hence is popular as an intercrop to enrich soil fertility. Although with immense genetic and morphological variabilities present for pod length, pod aroma, pod color, pod shape, and pod fiber, this orphan legume has not been given due emphasis. Few high-yielding determinate and indeterminate varieties have been produced which has changed the pattern of Indian bean cultivation from intercropping to monoculture. These released varieties are almost uniform considering pod qualities and unable to cater the needs of consumer preferences. Consumer preference varies from small, tubular, curved, light green-seed-filled pods to long, flat, strait, dark green/pigmented pods. Besides, this crop is predominantly used as an intercrop or kitchen garden crop. One can find the vines of this crop on almost every hut of tribal area or in the kitchen garden of urban homes. Despite its importance as a protein-rich vegetable in daily diet, the crop has not been given much importance as far as crop improvement and molecular breeding are concerned.

Indeterminate growth habit is predominant in germplasm accessions and landraces of Indian bean which allows the terminal shoot apex to remain in vegetative state. During the domestication of *Lablab*, the trait would have evolved due to selection for the continuous picking of vegetable pods. Determinate habit leads to switching of the terminal meristem into reproductive state which produces inflorescence [1, 2]. Determinate types are preferred for sole cropping, synchronous maturity, and mechanized harvesting or ease of manual harvesting. Determinate-type cultivars have compact growth habit, reduced branching, short internodes, accelerated flowering, high harvest index and synchronized maturity.

Growth habit exhibited dominance of indeterminate over the determinate type in Indian bean [3, 4]. Results of F_2_ populations indicated that growth habit is governed by three genes *GH*_*1*_, *GH*_*2*_ and *GH*_*3*_, of which, two are complementary with dominance of indeterminate growth habit [5]. Determinate growth habit and photoperiod-insensitive flowering are linked in Indian bean [3, 6]. No efforts have been made in Indian bean for molecular dissection of growth habit and flowering. It has been categorized as an underexploited pulse vegetable crop [7]. Knowledge on molecular pathways governing growth habit and photoperiod-responsive flowering is completely absent in literature for Indian bean. Functional aspects of its genome also remain unexplored due to unavailability of sequence data. Identification and characterization of genes responsible for growth habit in Indian bean will provide deep insight into the physiological and biochemical aspects of the trait and enable the breeders to select and bred the cultivars of determinate type more precisely with reduced time and cost.

Gene discovery using public sequence databases of model plants is useful especially for crops that do not have this information [8]. In the present study, locus responsible for growth habit in Indian bean has been isolated and characterized by using the information available in common bean through candidate gene approach. The *Arabidopsis TERMINAL FLOWER 1* (*TFL1*) gene has a unique effect on shoot apex architecture through different developmental stages [9]. *TFL1* acts as a repressor for floral initiation and maintains the inflorescence meristem through suppression of the expression of *APETALA1* (*AP1)* and *LEAFY (LFY)* [10–13]*.* Antagonistically, *FLOWERING LOCUS T* (FT) interacts with Basic Leucine Zipper Domain (bZIP) transcription factor *FLOWERING LOCUS D* (*FD*) to form a heterodimer that binds to the promoter of *APETALA1* (*AP1*) which activates flowering initiation [14, 15]. Regulation of growth habit has been studied intensively in the common bean [16, 17]. *PvTFL1y* is a homologue of *Arabidopsis TFL* controlling growth habit in common bean [16]. *PvTFL1y* was validated as the homologue of *TFL1* through functional complementation [17]. They found two unique variations responsible for determinacy at the *PvTFL1y* locus, a retrotransposon and splice site mutation. Many homologues for growth habit have been identified in soybean, *GmTFL1* [18]; in pea *PsTFL1a*, *PsTFLb* and *PsTFL1c* [19]; in faba bean *VfTFL1* [20] and in citrus *CsTFL* [21]*.* The structures of *AtFT* and *AtTFL* proteins have been previously compared to determine the cause of their opposite actions and their interactions with other proteins [22–28]. Importance of anion-binding pocket, external loop on the fourth exon and many key residues have been proposed to be playing key role in opposite activity of *AtFT* and *AtTFL*, of these, *FT* protein structure has been extensively studied. Yet, the actual mechanism responsible for their opposite actions and key binding pocket(s) involved in their interaction with other proteins are unclear. However, the protein structures of wild type and natural mutants of *TFL* have not been compared.

Study in the Indian bean with *PvTFL1y-*specific primers showed the association of this marker with growth habit [6]. The present study aimed to identify, characterize and validate a locus responsible for growth habit in Indian bean utilizing candidate gene approach coupled with sequencing and protein modelling. Two important binding pockets have also been proposed on the basis of geometrical and topographical property analysis. Our findings may enable the molecular dissection and modulation of growth habit in Indian bean through genome editing.

## Methods

### Plant material and phenotyping

**Fig. 1:**
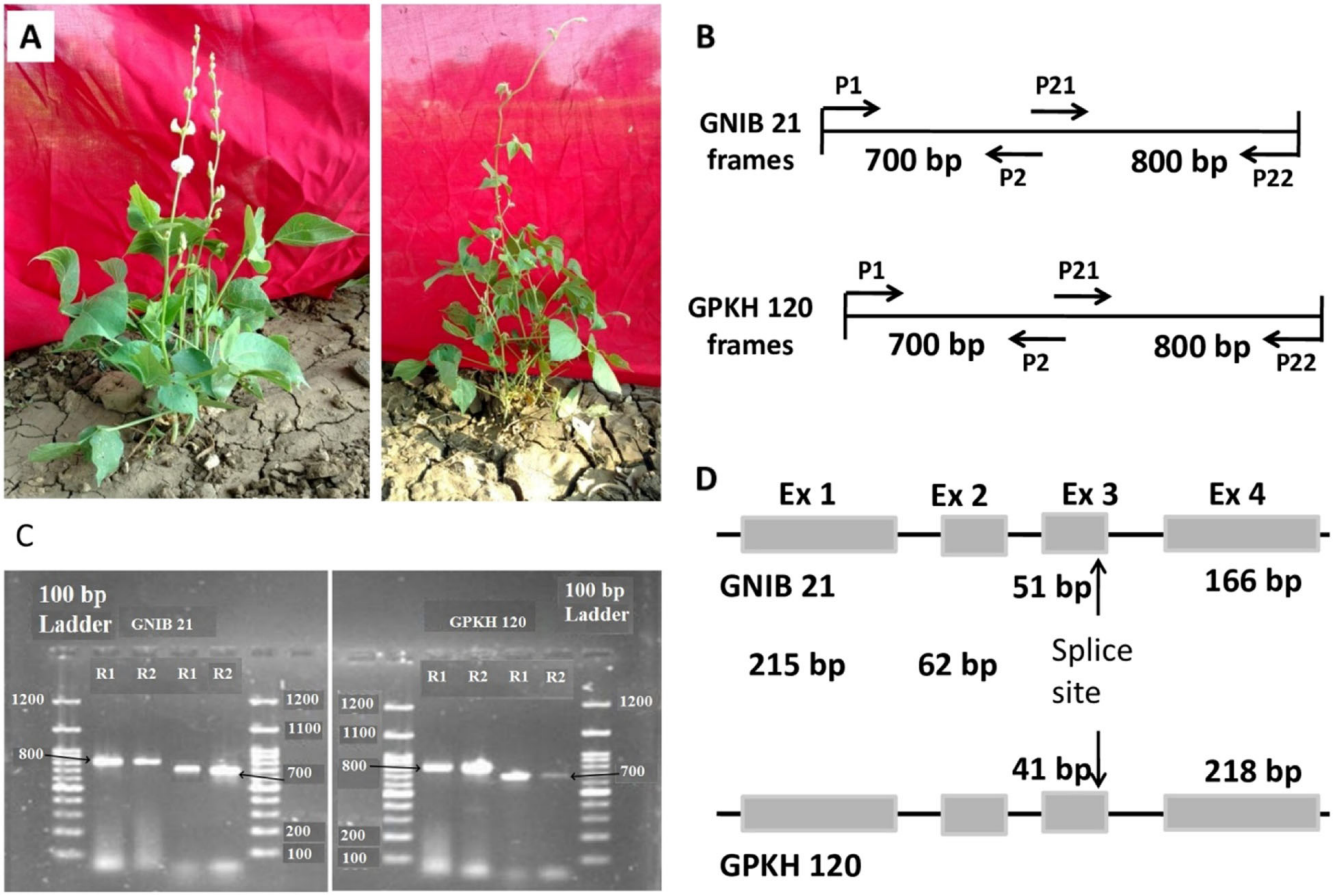
Phenotypic, molecular and allelic profiling of two Indian bean genotypes. **a** GNIB 21 (left, determinate) and GPKH 120 (right, indeterminate). **b** Annealing patterns of primers designed to amplify *TFL* in Indian bean, P_1_ and P_2_ are forward and revere primers for frame1, whereas, P_21_ andP_22_ are for frame2. **c** Amplification of *TFL* homologue in two frames from GNIB 21 (left) and GPKH 120 (right). R1 and R2 indicate replications. Amplicon lengths have been shown by black arrows. **d** Gene prediction in both the genotypes indicating splice site variation at the end of the third exon (Ex)

Two Indian bean genotypes, viz., GNIB 21 and GPKH 120 which are phenotypic extremes of growth habit were used for isolation of the *TFL* homologue (Fig. 1). GNIB 21 is a determinate cultivar, and GPKH 120 is an indeterminate cultivar. Plants were classified as determinate when the shoot apical meristem (SAM) acquires the identity of floral meristem which forms a terminal raceme, while in the case of indeterminate, main shoot axis continues a vegetative growth, never terminating into floral meristem. Eight unrelated indeterminate genotypes were utilized for validation.

### PCR amplification of candidate gene

To identify homologous sequence of *TFL* in Indian bean through candidate gene approach, the sequence was obtained from linkage study in Indian bean [6]. Basic Local Alignment Search Tool (BLAST) analysis showed its homology with the locus controlling flowering behaviour in common bean *PvTFL1y* (genotype G22833; accession JN418237.1)*.* The primers were designed in two frames from the sequence of *PvTFL1y* to isolate its homologue in Indian bean, expected to give an amplification of 700 and 800 bp fragments (Fig. 1c, Table 1). Genomic DNA was extracted from the young fresh leaves by cetyl trimethyl ammonium bromide (CTAB) method with some modifications. The target locus was amplified in two frames in both the cultivars using polymerase chain reaction (PCR) with *Taq* DNA polymerase (TaKaRa, Clontech, Japan). PCR mixture prepared in 200 μl contained approximately 100ng genomic DNA, 200 μM of dNTPs, 10 pmol of forward and reverse primers, standard *Taq* buffer (Mg^2+^ plus) and 1 unit of *Taq* DNA polymerase in total volume of 25 μl reaction. The PCR cycle involved 7 min of 95 °C initial denaturation and 35 cycles for 30 s at 94°C, for 45 s at 56°C and finally for 1 min at 72°C followed by 10 min of extension at 72 °C.

**Table 1:** Primers used for amplification of LprTFL locus

Primer	Sequences
1F^a^	5′TCGACTTGTATTCCTCACTCTCAC3′
2R^b^	5′TGCACAGACATCTAACAAGAATG3′
21F	5′CCCGCATAACTACCGGATTCT3′
22R	5′ACCAGGAGCATGAAGCTAGG3′

^a^*F* forward

^b^*R* reverse

### Sequencing and characterization

Amplified fragments from GNIB 21 and GPKH 120 were sequenced. The sequences obtained in two frames were merged, and overlapping sequences were identified in both directions using Bioedit Sequence Alignment Editor [29]. Merged sequences were then prepared in a single frame for both the parents. These sequences have been deposited to National Centre for Biotechnology Information (NCBI) database with Gene Bank accession number MK920414.1 and MK920413.1. Sequences obtained from these phenotypic extremes in relation to growth habit were aligned to identify the similarities and differences. Gene prediction was done with the help of Eukaryotic GeneMark.hmm version 3.54 [30] which revealed the size of probable exons and introns. Predicted sequences of four exons for nine genotypes including GNIB 21 were joined together to construct a full-length open reading frame, and protein sequences were predicted.

### Validation of allelic variation in germplasm lines

Genomic DNA was extracted from the young fresh leaves with CTAB method with some modifications from eight other indeterminate genotypes of Indian bean. Targeted fragment was amplified by using polymerase chain reaction (PCR) with *Taq* DNA polymerase (TaKaRa, Clontech, Japan). PCR mixture and cycle set up are the same as previously described. Whole *TFL* locus was amplified and sequenced from these genotypes. Gene prediction was done by GeneMark, and multiple sequence alignment of DNA as well as predicted protein sequences was carried out using ClustalW. The sequences have been submitted to NCBI database with GeneBank accession number MT230590 to MT230597.

### Protein modelling and topographical properties

The predicted protein sequences of GNIB 21 and GPKH 120 were subjected to SWISS-MODEL homology modelling online tool [31]. Proteins were modelled utilizing Protein Data Bank (PDB) entry 1wko.2.A (*Arabidopsis TFL1* protein) as reference. Quality of modelled protein was judged on the basis of GMQE (Global Model Quality Estimation) and QMEAN (Qualitative Model Energy Analysis) *Z*-score parameters [32, 33]. The best model was selected on the basis of GMQE score which ranges from 0 to 1, with a higher value indicating better reliability. The QMEAN *Z*-score provides an estimate of the “degree of nativeness” of the structural features observed in the model on a global scale. QMEAN *Z*-scores around zero indicate good agreement between the model structure and experimental structures. Scores of − 4.0 or below are an indication of models with low quality. The quality of the resulting models was also monitored with PROCHECK [34]. Geometrical and topographical properties of the modelled proteins were identified using Computed Atlas of Surface Topography of proteins (CASTp) 3.0 online server [35]. Molecular graphics images were produced using the UCSF Chimera package [36].

## Results

### Isolation of TFL homologue

In the present study, we used two genotypes, viz., GNIB 21 and GPKH 120 which are phenotypic extremes for growth habit. GNIB 21 possesses determinate growth habit, while GPKH 120 is indeterminate in nature (Fig. 1a). The main stem terminates into a raceme in the case of GNIB 21, while it never terminates into a flower in the case of GPKH 120. In common bean, few major and minor loci have been reported for growth habit, photoperiod sensitivity and flowering time [18, 37–42]. Findings showed that PvTFL1y is a functional homologue of TFL1 in common bean [17]. The primers designed from PvTFL1y in two frames were successful to amplify the TFL homologue (now referred as LprTFL : *Lablab purpureus* TFL) and yielded expected amplicons of 700 and 800 bp in GNIB 21 and GPKH 120, respectively (Fig. 1b, c, Table 1).

### Allelic characterization of LprTFL

Two amplicons obtained from each parent were sequenced; BLAST analysis indicated highest identity of 89.49 % with the TFL1y locus of common bean genotype G22833 (accession JN418237.1) with a zero *E*-value. The analysis revealed percent identity of 78.49%, 80.57%, and 82.00% with *Glycine soja*, *Glycine max*, and *Vigna* *unguiculata*, respectively. The automated gene structure prediction with GeneMark in both parental sequences revealed that the first and second exons of alleles are identical. However, length as well as end and start points of the third and fourth exons varied between the two alleles, respectively (Table 2). The length of the third and fourth exons in GPKH 120 is 41 bp and 218 bp, respectively. While, in the case of GNIB 21, the length of the third and fourth exon is 51 bp and 166 bp, respectively (Table 2).

**Table 2:** Gene prediction for *LprTFL* in ten genotypes of Indian bean using GeneMark

Genotype		Total length amplified (bp)	Exon type, range and length (bp)			
			Internal	Internal	Internal	Terminal
GNIB 21	R^a^	1387	11–225	414–475	955–1005	1156–1321
	L^b^		215	62	**51**^**c**^	**166**^**c**^
GP 189	R	1383	35–249	438–499	979–1019	1128–1345
	L		215	62	41	218
GP 1	R	1381	33–247	436–497	977–1017	1126–1343
	L		215	62	41	218
GP 167	R	1380	32–246	435–496	976–1016	1125–1342
	L		215	62	41	218
GPKH 120	R	1361	12–226	415–476	957–997	1106–1323
	L		215	62	41	218
IBGP 1	R	1386	33–247	436–497	977–1017	1126–1343
	L		215	62	41	218
IBGP 2	R	1383	34–248	437–498	978–1018	1127–1344
	L		215	62	41	218
IBGP 3	R	1384	33–247	436–497	977–1017	1126–1343
	L		215	62	41	218
IBGP 4	R	1390	40–254	443–504	984–1024	1133–1350
	L		215	62	41	218
IBGP 5	R	797	–	–	393–433	542–759
	L		–	–	41	218

^a^*R* range

^b^*L* length

^c^exon length differences in GNIB-21

**Fig. 2:**
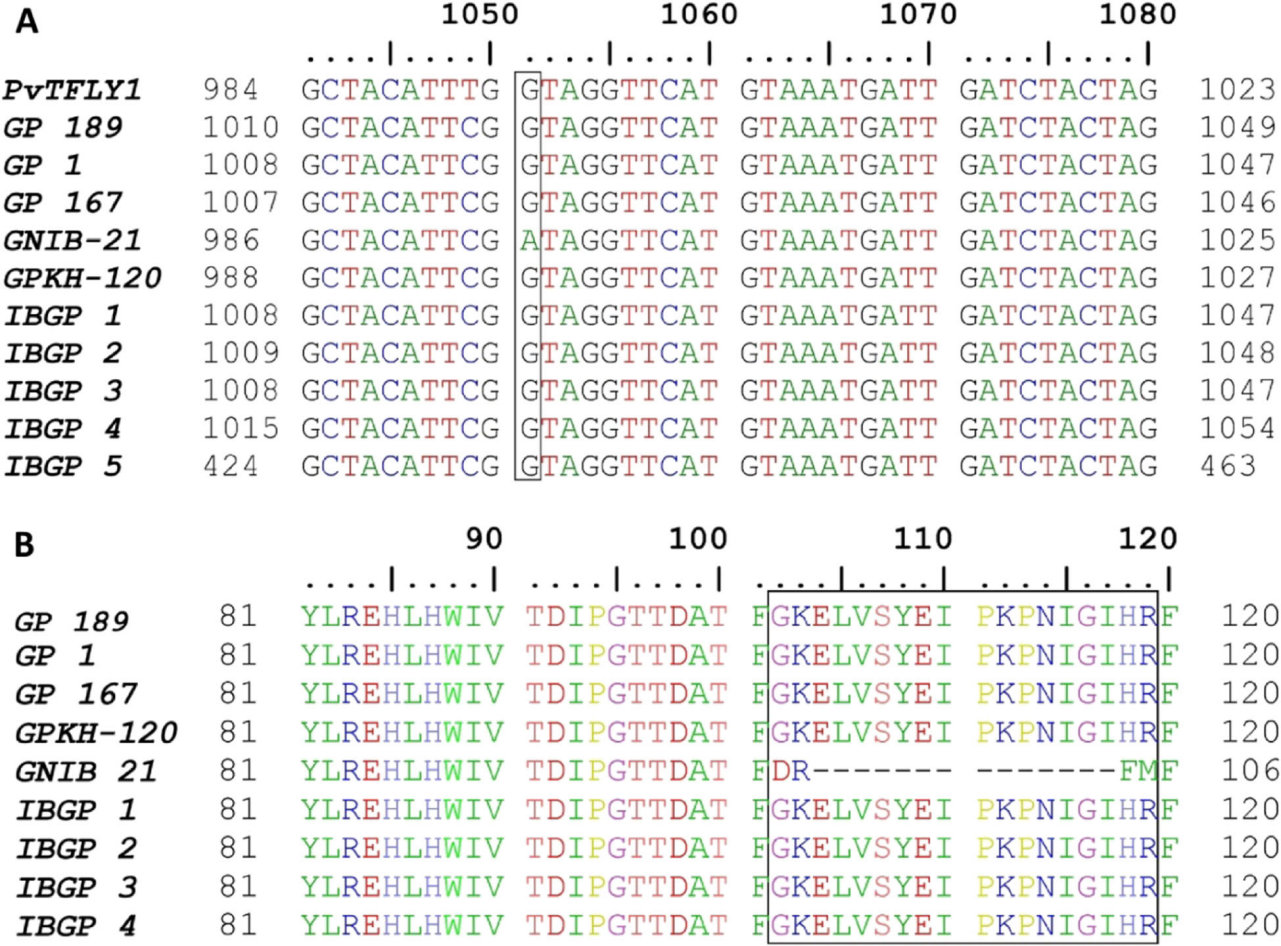
Multiple sequence alignment of Indian bean genotypes. GNIB 21 is a determinate type, and the remaining genotypes are indeterminate. **a** DNA sequence alignment of ten genotypes. Splice site transition from G to A creating SNP at the end of the third exon is shown by a rectangle. **b** Protein sequence alignment of nine genotypes of Indian bean and *PvTFL1y* of common bean. Fourteen amino acids (from position 104 to 117) missing and amino acid substitutions in GNIB 21 due to splice site transition are shown by a rectangle

The results indicated probable splice site variation at the junction of the third exon and third intron, which altered the length as well as the start and end points of the fourth and third exons, respectively. The sequences obtained from both the parents were compared using Bioedit tool for allelic characterization which revealed transition of G → A at the end of the third exon (Figs. 1d and 2a, Table 2). It was investigated through validation in germplasm lines that this transition of G → A is the main cause for the transformation of the shoot apical meristem from vegetative to reproductive architecture which forms a terminal flower in determinate parent GNIB 21.

### Validation of LprTFL in germplasm lines

Genotyping by sequencing approach was followed for validation of this candidate gene in eight indeterminate germplasm lines of Indian bean. The full-length sequences derived from GNIB 21, GPKH 120 and germplasm lines were merged on the basis of overlapping ends and aligned by ClustalW using MEGA. GeneMark was used to predict exon sequences of eight germplasm lines. As expected, all the indeterminate lines had guanine at the transition site at the end of the third exon confirming its involvement in growth habit differences (Fig. 2a). All the indeterminate genotypes possess exon sequences as well as lengths identical to GPKH 120 (Table 2). Predicted exon sequences of nine genotypes including GNIB 21 were used for prediction of translated protein. The protein alignment indicates absence of 14 amino acids (104 to 117) in GNIB 21 which might be the major cause of determinate growth habit by rendering the *TFL* protein non-functional (Fig. 2b). Apart from that, a non-synonymous substitution is apparent at the 119th position. The first frame was not amplified in genotype IBGP 5, so it was not included in protein alignment.

### Protein modelling and topographical properties

**Fig. 3:**
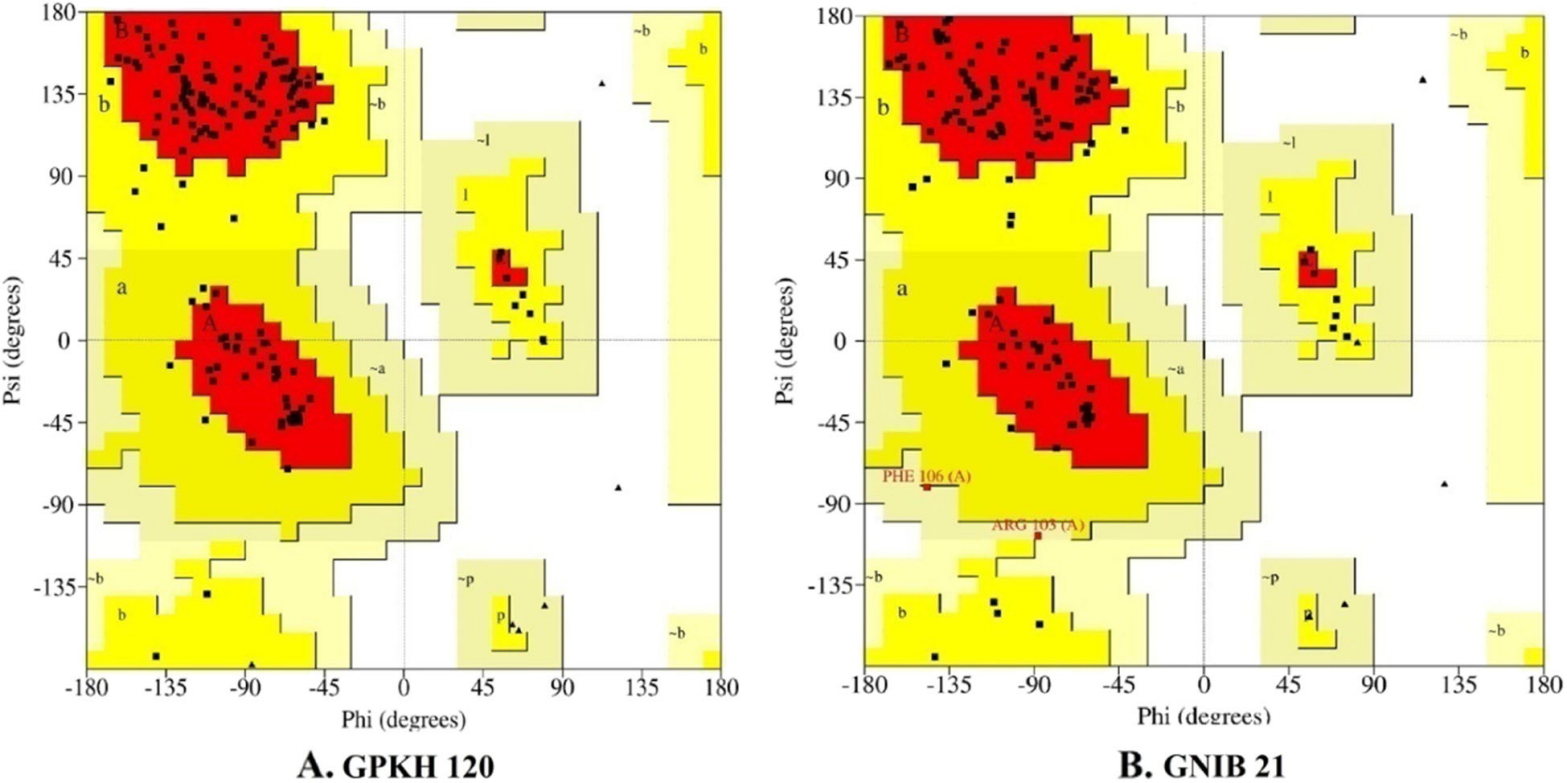
Ramchandran plots of modelled TFL proteins from two genotypes of Indian bean. Ramachandran distributions of amino acids in the model were calculated with PROCHECK

The structures of TFL proteins of two Indian bean genotypes were predicted using SWISS-MODEL homology modelling online server. GMQE (GPKH 120: 0.87 and GNIB 21: 0.80) and QMEAN Z (GPKH 120: 0.11 and GNIB 21 : − 1.57) scores indicated that these models possessed reliable and good quality. The quality of the resulting models was monitored with PROCHECK. Ramachandran plot analysis revealed that 87.7% of the non-glycine residues in the GPKH 120 TFL protein structure fell within the most favoured regions, with a further 12.3% in the additionally allowed region (Fig. 3a). No residues were in the generously allowed or disallowed regions. Similarly, the GNIB 21 TFL protein model comprised of 85.2% of non-glycine residues in the most favoured regions, while 13.8% and 1.6% in additionally and generously allowed regions, respectively (Fig. 3b). There was no residue found in the disallowed region.

**Fig. 4:**
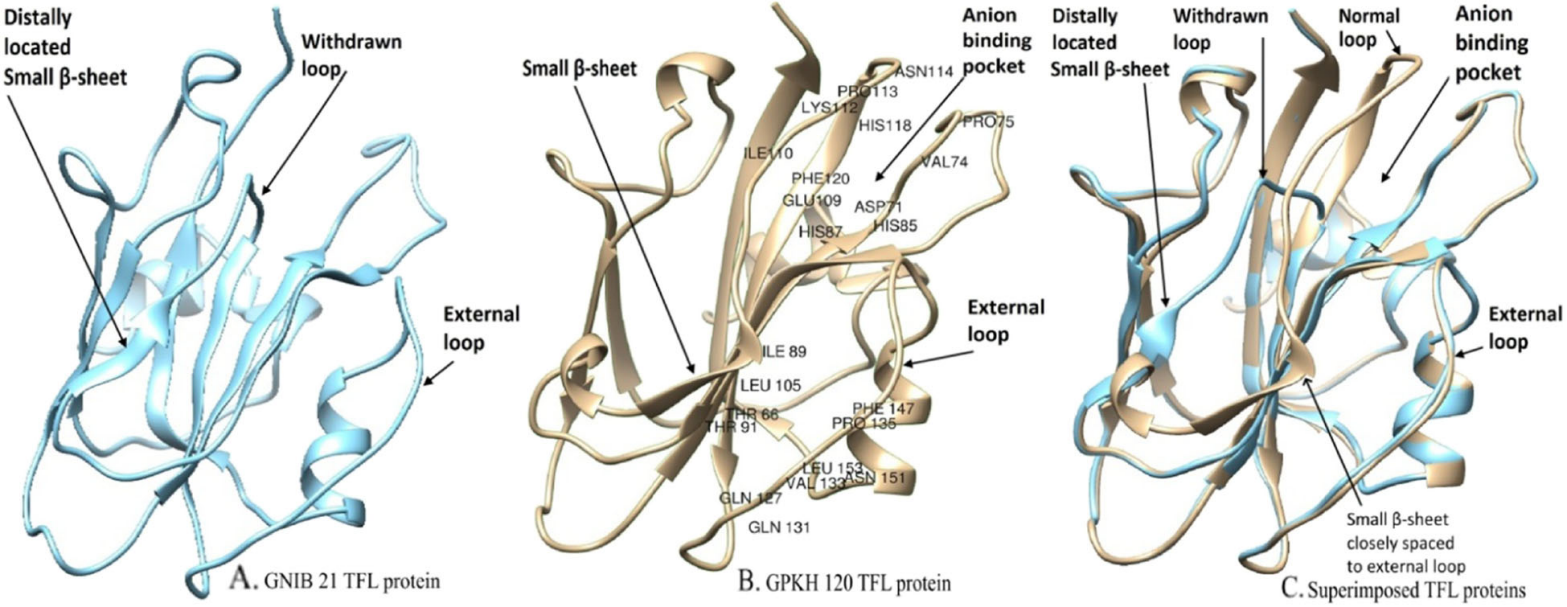
Comparison of predicted TFL protein structures of two Indian bean genotypes. **a** The withdrawn loop and distally located small β-sheet away from the external loop are apparent due to deletion of amino acids owing to splice site transition in GNIB 21. **b** Amino acid residues involved in the formation of binding pockets are depicted for GPKH 120 to narrate structural anomalies between two proteins. The normal loop and well-placed small β-sheet adjacent to the external loop provide wild type flowering suppression activity. **c** Superimposed wild type and mutant proteins depicts structural abnormalities related to anion-binding pocket and small β-sheet

Predicted TFL protein structures of GPKH 120 and GNIB 21 based on homology modelling are depicted in Fig. 4. The protein sequences of these two genotypes showed sequence identity of 74.57 and 72.33%, respectively, with template PDB entry 1wko.2.A (Arabidopsis TFL1 protein). Deletion of 14 amino acids owing to splice site variation in GNIB 21 corresponds to absence of extended normal loop made up of 104 to 117 amino acid residues (Fig. 4a). This deletion has resulted into shortening of one of the four major β-sheets. This anomaly has disrupted anion-binding pocket which has been previously proposed to play very important role in interaction of AtTFL with regulatory protein involved in plant growth architecture. The same loop is present in TFL protein of GPKH 120 with anion-binding pocket undisturbed (Fig. 4b). Apart from this, structural differences were also observed for residue positions from 99 to 105. Both deletion of 104 to 117 residues as well as substitutions of K103, H118 and R119 by R103, F118 and M119 might be responsible for protein structure anomalies.

Another anomaly was found for amino acid residues 99 to 105. This region is involved in formation of small β-sheet. This small β-sheet may be playing a very important role by interacting with neighbouring external loop (residue 130 to 141) encoded by the fourth exon (Fig. 4b, c). This small β-sheet is farther from the neighbouring loop in the case of GNIB 21, probably owing to the deletion and substitution found in the present study (Fig. 4a, c).

**Fig. 5:**
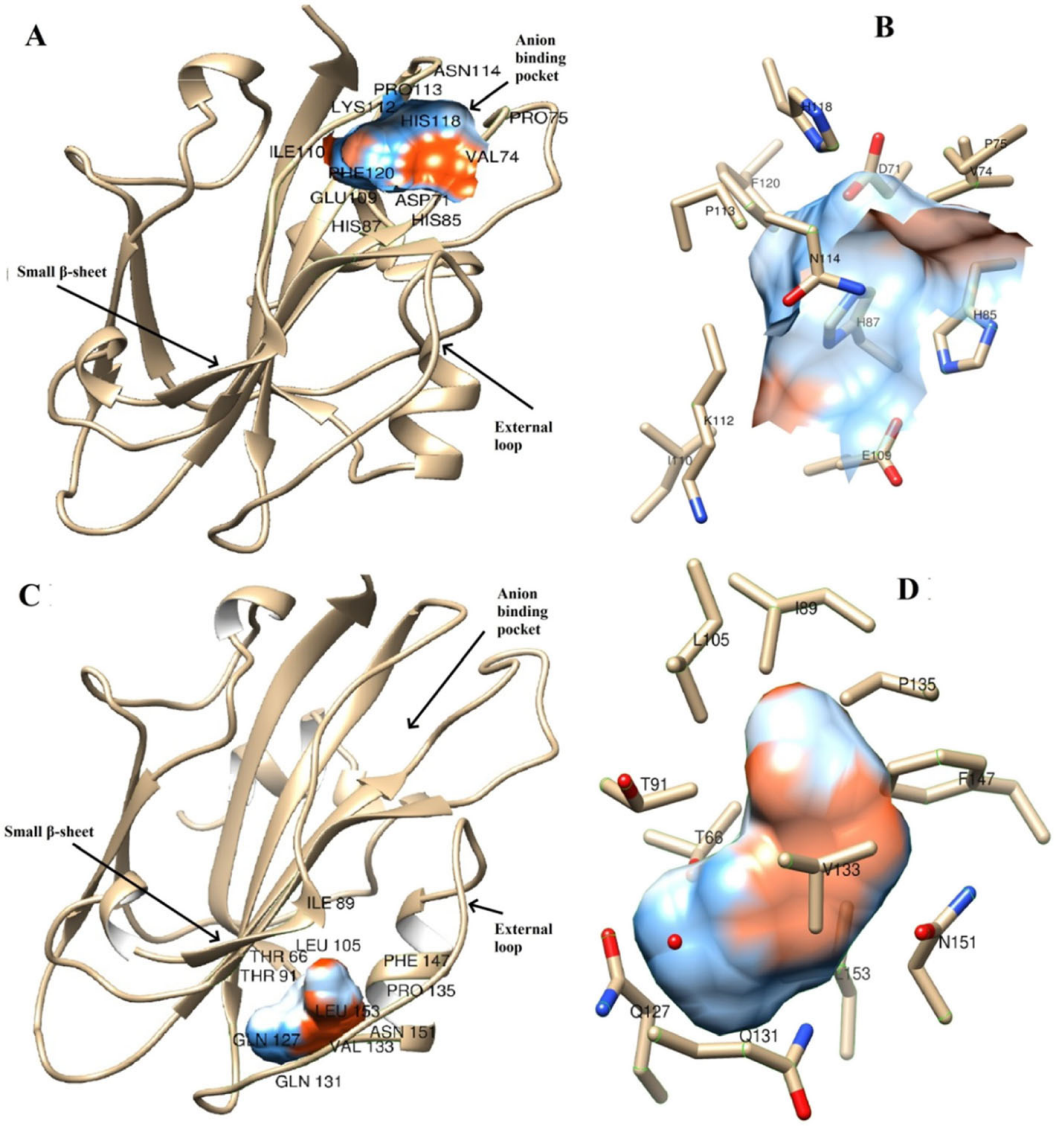
Predicted binding pockets of modelled TFL protein of GPKH 120 based on geometrical and topographical properties. **a** Predicted anion-binding pocket and the amino acid residues involved in its formation. **b** Close-up view of amino acid side chains involved in formation of anion-binding pocket. **c** Predicted a secondary binding pocket involving small β-sheet and external loop along with amino acid residues involved in its formation. **d** Close-up view of amino acid side chains involved in formation of a secondary binding pocket

Geometrical and topographical properties of the modelled proteins were identified using CASTp 3.0 online server. Functionally important residues located in the identified pocket (reported as anion-binding pocket previously in *AtTFL*) in *TFL* protein of GPKH 120 are ASP71, VAL74, HIS85, HIS87, GLU109, ILE110, LYS112, PRO113, ASN114, HIS118 and PHE120 (Fig. 5a, b). Apparently, this binding pocket was disturbed and not predicted by CASTp for GNIB 21 due to deletion of key amino acids. Another binding pocket was predicted for GPKH 120, created by proximity of small β-sheet and neighbouring external loop (Fig. 5c, d). The key amino acid present in small β-sheet, playing a role in the formation of this binding pocket, is LEU105. Other amino acid residues involved in the formation of this pocket are THR66, ILE89, THR91, GLN127, GLN131, VAL133, PRO135, PHE 147, ASN151 and LEU153. Of these, THR66, ILE89 and THR91 are situated in two centrally located large β-sheets, while residues GLN127, GLN131, VAL133 and PRO135 are located in a neighbouring loop (Fig. 5c, d). Other residues, viz., PHE 147, ASN151 and LEU153 are present in a small α-helix connected to and present beside the loop. This binding pocket was not predicted for GNIB 21 as the small β-sheet is no longer in proximity to the external loop due to deletion. These two binding pockets or any other significant binding pocket was not predicted for GNIB 21 by CASTp 3.0, owing to the absence of amino acid residues involved in the formation of the pockets or a structural anomaly created due to this deletion, rendering this protein non-interactive.

## Discussion

The major physiological constraint for pulse improvement is their indeterminate growth habit. Indeterminacy might have been selected during domestication due to pod pickings at regular intervals as well as plant’s escape from biotic and abiotic stresses due to continuous reproductive flushes. Most of the land races and cultivars of Indian bean are indeterminate in nature. Recently, Indian bean’s cultivation is shifting from intercropping to monoculture due to availability of determinate varieties. Determinate growth habit is preferred for monoculture due to the early flowering, photo-insensitivity, synchronous pod maturity, ease in manual harvesting, high harvest index and non-requirement of support system for plant growth.

Due to unavailability of genome sequence database, not a single economically important gene has been identified in Indian bean. Comparative gene mapping indicated that genomic structure of related plant species has been conserved in context to genetic content, order and function [43]. Phylogenetic analysis indicated a close evolutionary relationship between Indian bean, common bean and soybean [44]. *Arabidopsis TFL1* gene was found to have substantial effect on shoot apex architecture during various developmental stages [9]. In pea, two homologous loci were identified: *PvTFL1a* as the *Determinate* (*DET*) gene and *PvTFL1c* as the *Late Flowering* (*LF*) gene [19]. *PvTFL1y* locus in common bean is related with growth habit and cosegregated with determinacy locus [17]. Two orthologs of pea *TFL1a*, *GmTFL1a* and *GmTFL1b*, were isolated from the soybean through molecular dissection; mapping analysis indicated that *GmTFL1b* was a candidate for *Dt1* [45]*.* Cosegregation of the marker and *TFL* locus indicated that *LprTFL* is a homologue of the *TFL1* in Indian bean [6].

*Arabidopsis TFL1* gene belongs to *CETS* (Centroradialis/Terminal Flower 1/Self-Pruning) family which has an important role in the transformation of vegetative shoot apex into inflorescence morphology [46–48]. A splice site variation present at the end of the third exon of *LprTFL* is found to be responsible for growth habit difference in the present study. This loss of function splice site variation is SNP, created due to transition from guanine to adenine which results into determinate growth habit (Fig. 2a). Fourteen amino acids are found to be missing due to splice site transition in final predicted protein of determinate variety GNIB 21 (Fig. 2b). Florigen *FT* is involved in the transition from vegetative to reproductive phase and flowering, while *TFL1* negatively influences this transition [49]. The transition at splice site probably made *TFL* protein non-functional, unable to suppress termination of shoot apical meristem into floral architecture. Determinate growth habit in soybean is associated with four distinct SNPs in the *GmTfl1* gene, each of which led to a single amino acid change [18]. Two alterations in *PvTFL1y* locus, a retrotransposon and a *splice site mutation*, were responsible for recessive nature of *fin*, a determinacy locus of common bean [17]. A strong association of SNP was found with the determinacy trait in 142 pigeonpea lines [50]. A novel non-synonymous SNP in exon 4 of cowpea *TFL1* resulted from transversion of cytosine to adenine was found to be responsible for determinate growth habit [51].

A linkage relationship between growth habit and flowering homologues has been reported in soybean [52, 53], pea [19] and common bean [2]. Linkage between growth habit and photoperiod-responsive flowering has been reported in Indian bean [5, 6]. Two *FT* homologues *GmFT2a* and *GmFT5a* were found to be involved in the control of photoperiodic flowering in soybean [54]. The possibility of involvement of complicated CO-FT regulon in the photoperiod regulation of flowering time has been suggested in soybean [55]. *GmCOL1a/b* may serve as suppressors of photoperiodic flowering in soybean under long-day conditions by suppressing the florigens *GmFt2a/GmFT5a* in coordination with *FT* homologues [56]. Available reports indicate that *FT/TFL1* genes are major target of evolution in nature which shows 60% homology and encodes phosphatidylethanolamine binding proteins (PEBPs) [49]. This indicates that such CO-FT regulon might also exist in Indian bean, and *LprTFL* might be a very important component of this molecular pathway.

Deletion of 14 amino acids owing to splice site variation in GNIB 21 corresponds to absence of extended loop made up of 104 to 117 amino acid residues. This deletion has resulted into shortening of one of the four major β-sheets. This anomaly has disrupted anion-binding pocket which plays a very important role in the interaction of *TFL* with other regulatory protein involved in plant growth architecture. This potential anion-binding site has been previously proposed to play a key role in interaction of *TFL* protein with phosphorylated proteins [22]. The same loop is present in *TFL* protein structure of GPKH 120 with anion-binding pocket undisturbed (Fig. 4b, c). Apart from this, structural differences were also observed for residue positions from 99 to 105. Deletion of both 104 to 117 residues and substitutions of K103, H118 and R119 by R103, F118 and M119 might be responsible for this difference (Figs. 2 and 4b and a–c). These structural differences might have rendered GNIB 21 *TFL* protein non-functional, unable to suppress flowering. The fourth exon of *TFL* plays a very important role in the flowering inhibition of *Arabidopsis* [23]. They divided exon 4 into 4 segments and found that segment B (comprised of 17 residues from 128 to 145 positions) of exon 4 is playing a major role in the flowering inhibition by *AtTFL*. The structural anomaly found in the present study corresponds to segment A; however, the deletion in segment A has disturbed the anion-binding pocket. This suggests that segment A and segment B both are involved in the formation of ligand binding protein. The missing region of segment A owing to splice site variation is comprised of GLU109, ILE110, LYS112, PRO113, ASN114 and HIS118 residues which are involved in the formation of anion-binding pocket possibly by interacting with ASP71, VAL74, HIS85 and HIS87 as indicated by studies on geometrical properties using CASTp (Fig. 5a, b). Of these, HIS85, HIS87, GLU109 and HIS118 have been shown as very important residues for creating anion-binding pocket in *Arabidopsis FT/TFL* [23]. Even single substitution of the first two HIS residues have been shown to have an effect on flowering inhibition activity of *TFL* in *Arabidopsis* [24]. In the present study, these HIS residues are intact; however, other amino acid residues are absent due to deletion in GNIB 21. The residues situated in this deleted region must be necessary to form anion-binding pocket along with HIS85 and HIS87. Mutations in GLU109 confer *FT*-like activity to *TFL* in Arabidopsis [25]. Tyr85 of *AtFT* forms an intra-molecular bond with the Glu109 as part of a hydrogen bond network [26]. Their in vitro and in vivo studies indicated that FT-PC (phosphocholine) interaction is involved in flowering time control and plays a supplementary role in DNA binding and formation of complete flowering activation complex (FAC). R119 residue is a very important component contributing to the formation of anion-binding pocket. Owing to the structural identity of *FT* and *TFL*, substitution of R119 by M119 in mutant *TFL* protein of GNIB 21 might have also contributed to its non-functionality.

Geometrical and topographical properties of the modelled proteins were identified using CASTp 3.0 online server. Figure 5 shows the binding pockets of *TFL* proteins of GPKH 120. Functionally important residues are located in the identified pocket in *TFL* protein of GPKH 120 which corresponds to a previously reported anion-binding pocket of *Arabidopsis TFL* [23]. These residues are ASP71, VAL74, HIS85, HIS87, GLU109, ILE110, LYS112, PRO113, ASN114, HIS118 and PHE120 (Fig. 5a, b). Apparently, this binding pocket was disturbed and not predicted by CASTp for GNIB 21 due to deletion of key amino acids. The importance of this pocket in binding with bZIP transcription factor has been demonstrated [14, 15, 23, 26–28, 57]. Our in silico analysis supports involvement of anion-binding pocket for protein–protein interaction between *TFL* and other interactors ultimately leading to flowering inhibition or indeterminate growth habit.

Another anomaly was found for amino acid residues 99 to 105. This region is involved in the formation of small β-sheet (Fig. 4a–c). This small β-sheet may be playing a very important role by interacting with a neighbouring external loop (residues 130 to 141) present on segment B encoded by the fourth exon. The contrasting effect of *FT* and *TFL* on flowering may be due to the difference in the structure of this loop [23]. This β-sheet is longer, located farther away from and unable to interact with external loop of segment B in case of GNIB 21, probably owing to the deletion and substitution found in present study (Fig. 4a, c). In the case of GPKH 120, this binding pocket consists of LEU105, the only amino acid of small β-sheet playing role in formation of the pocket. Other amino acid residues involved in formation of this pocket are THR66, ILE89, THR91, GLN127, GLN131, VAL133, PRO135, PHE 147, ASN151 and LEU153. Of these, THR66, ILE89 and THR91 are situated in two central large β-sheets, while GLN127, GLN131, VAL133 and PRO135 are located on a neighbouring loop. Other residues PHE 147, ASN151 and LEU153 are present in a small α-helix connected and present beside the external loop of segment B. The structural differences in this external loop are most prevalent at residue positions 132–139, indicating the possibility of molecular surface that may act independently from anion-binding site [23]. The variable part of this loop is also adjacent to the 60–66 loop, another region of variability between *FT* and *TFL1* [23]. All these amino acid residues are intact in the *TFL* protein of GNIB 21 except LEU105. Here, we propose that Leu105 may be playing a role in the formation of molecular surface for secondary binding site independent of anion-binding pocket in conjunction with external loop of segment B. In nutshell, deletion in GNIB 21 has resulted in displacement of small β-sheet away from external loop, disturbing the binding pocket rendering *TFL* protein non-functional.

We envision that a loop present on segment B and small β-sheet are together involved in the interaction of *TFL* protein with other proteins. Either any of these two structural anomalies or both the anomalies might be responsible for the non-functionality of *TFL* protein in GNIB 21. Both the anion-binding pocket and the external loop imparts functional specificity to *FT* and *TFL* proteins [23]. Both these sites may jointly be responsible for interaction with other protein or have independent interactions with a single protein or two different proteins. The possibility of interacting with multiple proteins may not be overlooked as *TFL* is commonly involved in different flowering pathways from autonomous to environmentally induced. Multiple proteins that interact with *TFL* have been identified [14, 15, 58]. Possibly, adjacent external loop present in segment B may contribute to the periphery of a protein–protein interface centred on the anion-binding pocket [23]. One major candidate interacting with *TFL* protein is bZIP transcription factor encoded by flowering locus D (FD). FD is involved in both positive and negative regulation of flowering through formation of phosphorylation dependent complex with *FT* or *TFL1* [27]. *TFL1* protein is capable of interacting with the FD transcription factor, *tfl1* mutants flower early and their shoot apical meristem is converted into a terminal flower [59]. The binding pockets identified in present study may be playing an important role for facilitating *TFL* protein’s interaction with bZIP transcription factor FD [14, 15, 23, 26–28, 57] or 14-3-3 like protein of Indian bean [26–28] or an unknown ligand [25, 60] or other protein interactors. Apart from GNIB 21 and GPKH 120, we have utilized eight other indeterminate genotypes for validation of splice site SNP. Inclusion of more determinate and indeterminate genotypes may have provided better confirmation about involvement of this SNP in governing growth habit. However, the deletion of 14 amino acids caused by splice site SNP disturbs previously identified anion-binding pocket (Fig. 4c). This provides additional proof that splice site transition reported in present study is responsible for growth habit differences in Indian bean.

## Conclusion

Allelic characterization of genes responsible for growth habit and photoperiod-responsive flowering may throw deeper insight into molecular mechanisms responsible for shaping plant architecture and enable modulation of these traits through genome editing. A splice site SNP in *TFL* locus is responsible for determinate growth habit in GNIB 21. This splice site variation result into variation in the length of the third and fourth exons which eventually leads to deletion of 14 amino acids in final protein sequence. This structural anomaly disturbs previously reported anion-binding pocket and secondary binding pocket due to displacement of small β-sheet away from external loop rendering *TFL* protein non-functional. Recently, Clustered Regularly Interspaced Short Palindromic Repeats (CRISPR)/Cas9-mediated targeted mutagenesis of *GmFT2a* has been demonstrated in soya bean which delayed flowering time [61]. Our study indicates that such kind of paradigmatic manipulation of the plant architectural traits would also be possible by disrupting the splice site or by simply creating a single base substitution at this junction. The study also emphasizes that in silico analysis of structural differences in wild type protein and its natural non-functional variant may give better insight about key structural features.

## Data Availability

The dataset supporting the conclusions of this article are included within the article. The sequencing data have been submitted to the NCBI repository.

## Abbreviations

TFL: Terminal flowering locus
SNP: Single-nucleotide polymorphism
GH: Growth habit
FT: Flowering locus T
BLAST: Basic Local Alignment Search Tool
PCR: Polymerase chain reaction
UCSF: University of California, San Francisco
NCBI: National Centre for Biotechnology Information
MEGA: Molecular Evolution Genetics Analysis
CASTp: Computed Atlas of Surface Topography of proteins
PDB: Protein Data Bank
bZIP: Basic Leucine Zipper Domain
CRISPR: Clustered Regularly Interspaced Short Palindromic Repeats
Cas: CRISPR-associated protein
Ex: Exon
